# Swallowing Grief: Medication, Memory, and Relapsing

**DOI:** 10.1192/j.eurpsy.2026.11413

**Published:** 2026-07-31

**Authors:** A. Mančić, D. Polić, G. Rubeša, T. Grahovac Juretić

**Affiliations:** 1 Department of Psychiatry, Clinical Hospital Center Rijeka; 2 Department of Psychiatry, Teaching Institute of Public Health of Primorsko-Goranska County, Rijeka, Croatia

## Abstract

**Introduction:**

Psychotic depression is a severe and recurrent condition in which episodes are often triggered by psychosocial stressors. The role of symbolic meaning in treatment—such as the transference to a specific medication or the significance of traumatic anniversaries—may play an important role in relapse. This case highlights the complex interplay between pharmacological treatment, traumatic memory, and symbolic associations.

**Objectives:**

To illustrate how medication transference and the emotional weight of significant anniversaries may contribute to the recurrence of psychotic depression, despite long-term remission.

**Methods:**

A 59-year-old woman presented to the psychiatric emergency service with a two-week history of worsening symptoms: insomnia unrelieved by hypnotics, a sensation of being unable to swallow due to a sticky tongue, difficulty eating, gastric discomfort, inner restlessness, fear of loss of functionality, fear of upcoming daylight-saving changes, and a loss of her usual daily rhythm. She reported feeling detached from herself and unable to function.

**Results:**

The patient had experienced a single previous episode of psychotic depression in 2016, following her son’s suicide. At that time, her symptoms included withdrawal, insomnia, and swallowing difficulties similar to the current episode. She remained in stable remission for nine years on paroxetine 30 mg, venlafaxine 75 mg, and olanzapine 10 mg. At a recent follow-up, olanzapine was reduced to 5 mg due to weight gain. Within two months she relapsed. Exploration revealed that her son’s birthday had occurred 10 days earlier, and the tenth anniversary of his death was approaching. Importantly, her son had also been prescribed the same brand of olanzapine at the time of his death. For the patient, this medication appeared to function as a transitional object, maintaining a symbolic connection to her son. Despite weight concerns, she was reluctant to discontinue the drug, resumed 10 mg, and was motivated to increase to 15 mg. After reinstatement, her physical symptoms and sleep improved rapidly, while psychotic features diminished but did not fully remit.

**Conclusions:**

This case illustrates how psychiatric medications may acquire symbolic meaning and serve as transitional objects, especially in the context of unresolved trauma. The interplay of medication symbolism and traumatic anniversaries may contribute to relapse even after long remission. Greater awareness of these dynamics could enrich clinical understanding and guide more sensitive treatment planning.

**Disclosure of Interest:**

None Declared

